# Additive-Free Multiple Processing of PLA Pre-Consumer Waste: Influence on Mechanical and Thermal Properties

**DOI:** 10.3390/polym17162164

**Published:** 2025-08-08

**Authors:** Aleksandra Nešić, Rebeka Lorber, Silvester Bolka, Blaž Nardin, Branka Pilić

**Affiliations:** 1 Faculty of Technology Novi Sad, University of Novi Sad, Bulevar cara Lazara 1, 21000 Novi Sad, Serbia; brapi@uns.ac.rs; 2 Faculty of Polymer Technology, Ozare 19, 2380 Slovenj Gradec, Slovenia; rebeka.lorber@ftpo.eu (R.L.); silvester.bolka@ftpo.eu (S.B.); blaz.nardin@ftpo.eu (B.N.)

**Keywords:** bioplastics, poly(lactide), industrial waste, mechanical recycling, thermal properties, mechanical properties

## Abstract

Poly(lactide) (PLA) is the most versatile biopolymer with few possible end-of-life scenarios, like recycling, biodegradation/composting, and incineration. Biodegradation occurs under strictly defined conditions, and ultimately, PLA is landfilled, where it behaves like conventional plastics. To completely utilize the potential of PLA, it is necessary to increase the recycling and upcycling rates. In this work, the influence of 10 cycles of reprocessing PLA pre-consumer industrial waste on the material’s properties was examined. The mechanical milling of the material was followed by injection molding, and after each cycle, mechanical, thermal, chemical, and optical properties were studied. Between the cycles, no virgin PLA or any additives were added to enhance the properties. Results showed a slight decrease in molecular weight, while the thermal properties remained unchanged compared to the starting material.

## 1. Introduction

The growing demand for environmentally sustainable materials has driven the increasing adoption of bio-based polymers, among which poly(lactide) (PLA) has emerged as a leading alternative to petrochemical plastics. According to the market analysis carried out by Eureopean Bioplastics [1], PLA is the top produced bio-based and biodegradable polymer with a share of around 37% in 2024, with an estimated increase up to 42% in 2029. Derived from renewable resources such as corn starch and sugarcane, PLA offers advantages including biodegradability, biocompatibility, and favorable mechanical and thermal properties, making it widely used in single-use packaging, textiles, 3D printing, and biomedical applications [1,2,3,4].

Despite its ecological appeal, the environmental benefit of PLA depends largely on how it is managed at its end-of-life (EoL). PLA is compostable under industrial conditions (≥58 °C, high humidity, microbial activity), yet its biodegradation in natural or home composting environments is extremely slow, often requiring months to years [5,6]. In practice, a significant share of PLA ends up in landfills or incineration, undermining its circular potential [7]. Therefore, viable recycling strategies are critical for realizing the full sustainability of PLA.

Several EoL routes are available for PLA, including mechanical recycling, chemical depolymerization, industrial composting, and incineration with energy recovery [8,9,10,11,12,13,14,15,16,17]. Among these, mechanical recycling is recognized as the most economically and energetically feasible pathway in the short term, as it allows for the reuse of the polymer without depolymerization, maintaining the material’s structural integrity through physical reprocessing steps such as shredding, melting, and extrusion [18,19]. Mechanical recycling also aligns well with circular economy goals, especially in industrial and post-consumer contexts such as 3D printing and food packaging.

However, repeated mechanical recycling of PLA might introduce thermal and hydrolytic degradation, primarily through chain scission, leading to a reduced molecular weight, diminished mechanical properties, and altered viscosity [20]. Pillin et al. [21] investigated the influence of seven cycles of repeated injection molding on mechanical, rheological, and thermal properties of PLA, together with the effect of the addition of the stabilizers tropolone and quinone. They concluded that quinone is effective in maintaining PLA chain length, while pure PLA is moderately recyclable. Beltran et al. [22] examined the recyclability of PLA films after aging treatment and compared the influence of a washing step on recycled PLA properties, where the observed degradation of PLA did not influence the thermal and mechanical stability of the recycled compared to starting material. Scaffaro et al. [23] investigated the effect of five subsequent extrusion cycles on properties of PLA and PLA-based nanocomposites containing modified and unmodified hydrotalcite, where chain scission and a decrease in intrinsic viscosity was observed. In contrast, the effect on thermo-mechanical properties was not significant, especially within nanocomposites. Reprocessing and evaluation of properties of obtained films from recycled material after each recycling cycle was performed by Hidalgo-Carvajal et al. [24] using PLA 3D printing waste of a known origin and mixed PLA waste from personal protective equipment from COVID-19 in Spain. Waste of a known origin underwent five repeating cycles and mixed PLA waste underwent four repeating cycles, and results showed an increase in intrinsic viscosity and a significant decrease in molecular weight (48% for waste of a known origin and around 40% for mixed waste after the fifth reprocessing cycle). However, at the same time, the mechanical properties of the obtained films were not significantly changed. Reprocessing of PLA with a high MFI was investigated by Ramos-Hernandez et al. [11], where they also observed a significant decrease in MFI which was suppressed by the utilization of Joncryl^®^ as a chain extender. The possibility of recycling filaments for 3D printing was examined by Lee at al. [25] and they made five consecutive cycles. Due to the high standard deviations of results of mechanical testing they observed, they proposed only up to three recycling cycles. Six reprocessing cycles which consist of hot melt extrusion and injection molding of PLA and PLA-based composites loaded with fibers were carried out by Finnerty et al. [19], and in this work, the authors observed color change in specimens (from light to dark yellow) with the increasing of the number of cycles, and also a decrease in the mechanical properties, especially those of fiber-loaded composites due to the destructed morphology of the fibers. Żenkiewicz et al. [26] examined mechanical and thermal properties of PLA after 10 consecutive times of extrusion processing and obtained similar results of increasing MFI and a slight decrease in mechanical properties.

The decrease in the chain length due to hydrolysis restricts the recyclability of PLA, particularly for applications demanding high mechanical performance.

To mitigate such degradation, researchers have developed various strategies:

- Chain extenders, including epoxy-functionalized additives like Joncryl^®^ ADR, have been shown to restore molecular weight, increase viscosity, and stabilize mechanical properties over multiple cycles [27,28].
- Blending recycled PLA with virgin material (typically 30–70%) maintains mechanical integrity while enhancing sustainability [7].

In this work, pre-consumer waste underwent 10 processing cycles and properties were studied after each to better understand the influence of reprocessing on mechanical, thermal, chemical, and optical properties of PLA compared to the starting material.

## 2. Materials and Methods

Stramex PET d.o.o. (Podplat, Slovenia) supplied PLA bottles (pre-consumer industrial waste). They were used as received, without any treatment.

### 2.1. Processing of the Material

Bottles were milled using a Wanner milling machine for thermoplastics extruded on the twin-screw extruder Labtech LTE 20-44 (Labtech Engineering Company, Ltd., Bangkok, Thailand). The screws had diameters of 20 mm, an L/D ratio of 44:1, the screws rotated at 600 rpm, and the temperature profile was from the hopper (165 °C) to the die (180 °C).

**Figure 1:**
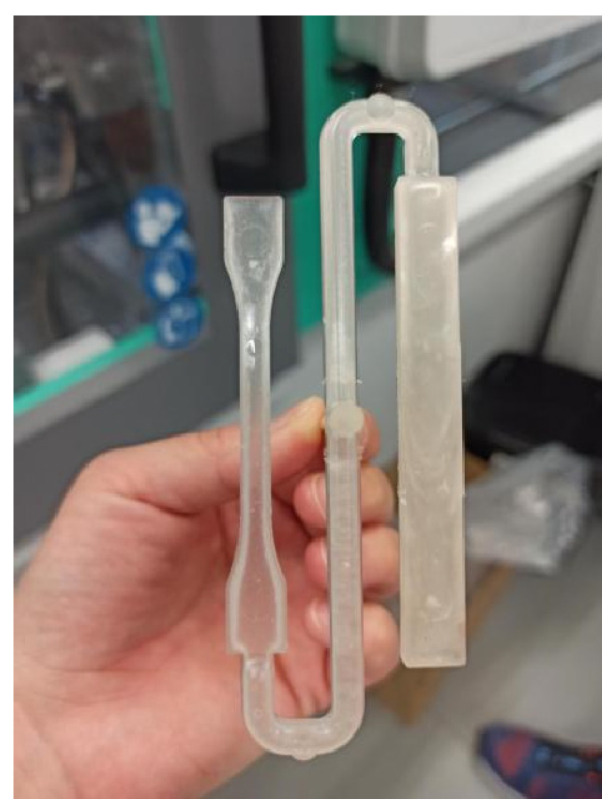
The item obtained by injection molding dedicated to the testing of mechanical properties of the material.

After extrusion, PLA pellets were processed using an injection molding machine (ARBURG Allrounder 320 C500-100 Golden Edition, Lossburg, Germany) using a specially constructed tool with dimensions according to the requirements of ISO standards for mechanical testing. The finished item obtained by injection molding is illustrated in Figure 1. The temperature was increased from the hopper (165 °C) to the die (180 °C). The tool temperature was set to 45 °C and the cooling time was set to 20 s. After the first cycle of injection molding, molded samples were milled and molded in nine more consecutive cycles. From each cycle, 10 samples from the middle of the process, when it is considered stable, were taken for further examination. Samples were completely uniform and defect free. The left part was used for tensile testing, while rectangular part was used for DMA and flexural testing. Between the cycles, milled material was dried at 75 °C until the moisture content was below 0.025% which was determined using the moisture analyzer HX204 (Mettler Toledo, Greifensee, Switzerland). No plasticizer or any other additive was added to PLA during the recycling.

### 2.2. Characterization of the Material

The melt flow index (MFI) of samples was evaluated according to ISO 1133 [29] via an MFI indexer (Dongguan Liyi Test Equipment, type LY-RR, Dongguan, China) at a temperature of 210 °C with a 2.16 kg weight. Results are expressed as an average value with standard deviation.

Thermal measurements were carried out with a differential scanning calorimeter (DSC 2, Mettler Toledo, Greifensee, Switzerland) under a nitrogen atmosphere (20 mL/min). The sample temperature was increased from 0 to 200 °C at a heating rate of 10 °C/min and held in the molten state for 5 min to erase their thermal history. After being cooled at 10 °C/min, the samples were reheated to 200 °C at 10 °C/min. The crystallization temperature (Tc), crystallization enthalpy (∆Hc), melting temperature (Tm), and melting enthalpy (∆Hm) were obtained from the cooling and the second heating scan. Moreover, the relative crystallinity values (Xc) were calculated using following equation:

(1) $$Xc\left(\%\right)=\frac{∆Hm-∆Hcc}{∆Hf}\times 100$$

where ΔHm refers to the enthalpy of melting;

ΔHcc refers to the enthalpy of cold crystallization;

ΔHf refers to the enthalpy of melting of 100% crystalline PLA and equals 93.6 kJ/g.

Thermogravimetric analyses (TGAs) were performed on a TGA/DSC3 (Mettler Toledo, Switzerland). The analyses were carried out in a nitrogen atmosphere (20 mL/min) from 40 to 600 °C with a heating rate of 10 °C/min, followed by a segment in the oxygen atmosphere (20 mL/min) from 600 °C to 700 °C while the heating rate remained the same.

Thermo-mechanical properties were examined using DMA 8000 (Perkin Elmer, MA, Shelton, CT, USA). The samples were heated at 2 °C/min from room temperature to 170 °C under an air atmosphere. A frequency of 1 Hz and an amplitude of 20 μm were used in dual cantilever mode.

Impact tests were performed on the pendulum instrument type LY-XJJD5 (Dongguan Liyi Test Equipment, Dongguan, China), according to ISO 179 [30]. The distance between supports was 60 mm, and a 2 J pendulum was used.

Flexural and tensile tests were conducted on the AG-X plus (Shimadzu, Kyoto, Japan) according to ISO 178 [31] and ISO 527-1 [32], respectively. Five measurements were performed for each sample.

Chemical properties were examined via infrared spectroscopy with Fourier transformation IRAffinity-1S (Shimadzu, Kyoto, Japan), measuring the transmittance (T%), in the wavenumber range of 4000–400 cm^−1^ and with 4 cm^−1^ resolution.

Crystallinity was determined using an X-ray diffractometer (XRD) MiniFlex 600 (Rigaku, Tokyo, Japan), using Cu-Kα radiation. The 2θ range was from 0 to 50, with a resolution of 0.04, and a 3 s hold.

Surface properties of materials were determined by measuring the contact angle against water (Osilla Goniometer, Sheffield, UK). One drop of distilled water was placed on the surface of the material, and the contact angle was determined using Osilla Contact Angle version 4.1.5. built-in software.

The colorimetric properties of the samples, subjected to multiple recycling cycles, were evaluated using the CIELAB color space system (CIELAB, Vienna, Austria), comprising parameters L*, a*, b*, c*, and h*. These parameters provide a comprehensive representation of the material’s perceived color. L* denotes lightness (0 for black, 100 for white), a* represents the red-green axis (positive for red, negative for green), b* represents the yellow-blue axis (positive for yellow, negative for blue), c* signifies chroma or saturation, and h* indicates the hue angle. A 3nh NR60CP spectrophotometer (Guangzhou, China) was used for evaluation of described parameters. ΔE* value, a color difference between the sample and the refference, was calculated using equation:

(2) $${∆E}^{*}=\sqrt{{\left({∆L}^{*}\right)}^{2}+{\left({∆a}^{*}\right)}^{2}+{\left({∆b}^{*}\right)}^{2}}$$

where ΔL* is difference in L* values between sample and reference;

Δa* is difference in a* values between sample and reference;

Δb* is difference in b* values between sample and reference.

## 3. Results and Discussion

**Figure 2:**
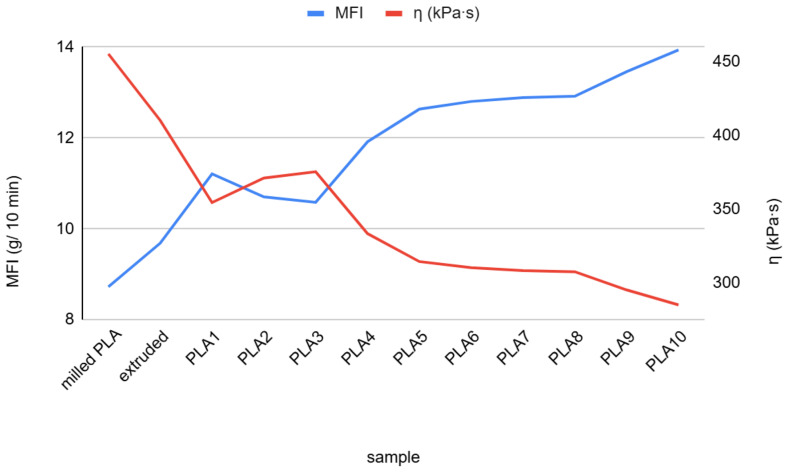
MFI (blue line) and viscosity (red line) of recycled PLA.

Figure 2 illustrates changes in MFI and viscosity with the consecutive processing of PLA. Except for cycles 2 and 3 (samples PLA2 and PLA 3), MFI is increasing, while the viscosity of the melt is decreasing. Since no processing additives were added to the PLA, these changes are a direct consequence of consecutive milling and injection molding, which induced chain scission and a decrease in the molecular weight of the PLA molecules.

**Figure 3:**
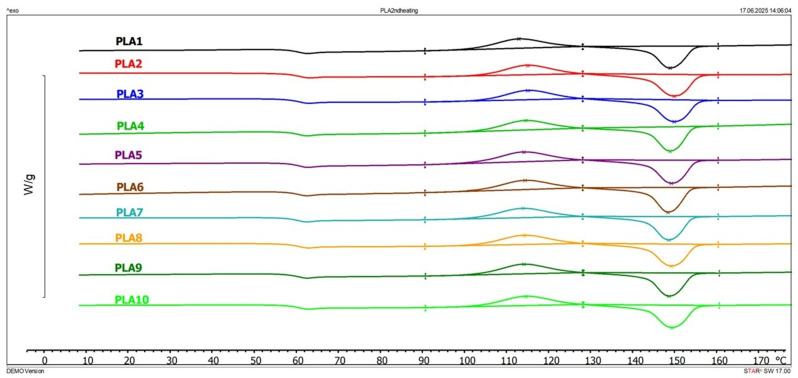
Thermograms of the second heating cycle.

Thermal properties of the examined samples are summarized in Table 1 and the thermograms of the second heating cycle are given in Figure 3. The present results are from the second heating cycle. It can be seen that there are no changes in Tg, Tc, and Tm; they are very constant with low deviation from the first to the last cycle. There are changes in the values of both crystallization and melting enthalpies, which do not follow the regular trend of change. Variations in enthalpies might be a consequence of the different crystallization times of the samples, since the processing was not performed the same day for all cycles. Xc, as a measure of relative crystallinity calculated from the enthalpies of cold crystallization and melting, varies from cycle to cycle. This is a consequence of both the decrease in the molecular weight of PLA molecules and the crystallization time of the samples. Samples sometimes underwent few cycles per day which does not allow for the complete cooling of the samples and complete crystallization.

The degradation temperature (Td_1_) was determined by the TGA method. The values have an almost linear trend of decreasing from 370 °C to 363.7 °C (Table 1), which is in line with MFI results. When the MFI is higher, molecular weight is lower, and it is expected that within corresponding samples, Td_1_ will be lower. Even though the degradation temperature is decreasing, the maximum difference is below 7 °C, which is only 2%. Considering DSC and TGA data, it can be concluded that thermal stability did not significantly change with multiple consecutive processings of PLA.

**Figure 4:**
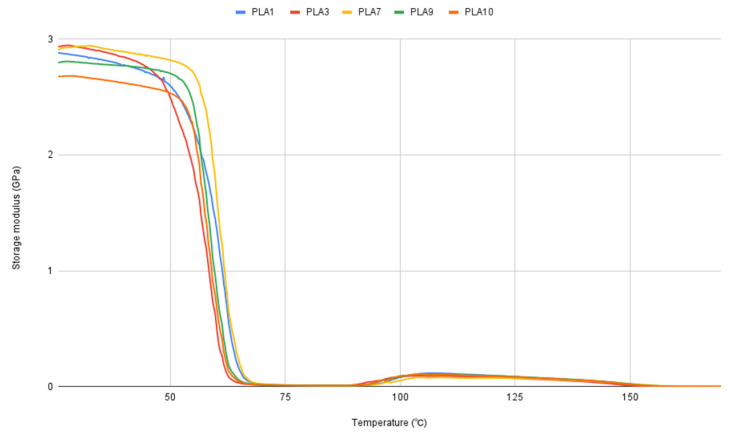
DMA curves of selected samples.

Dynamic-mechanical analysis results give insight into stiffness, storage modulus, and mechanical Tg values, which reflect mechanical softening (segmental motion). The difference in Tg values is low, less than 1 °C, which is in accordance with DSC results. The difference in Tg values determined by DSC and DMA (Table 2 and Figure 4) is expected, since the methods deliver different types of Tg. DSC is the best for thermodynamic Tg, which is related to the processing and the thermal stability of the material, while DMA gives insight into mechanical Tg, which is related to the functional behavior and the mechanical material’s design. DMA Tg has a higher value, and the expected difference compared to DSC Tg is from 5 to 20 °C, which is proven in this case, where DMA Tg has a higher value for 6 to 7 °C. Samples PLA5, PLA6, and PLA8 have two, and PLA7 has three crystallization peaks, which refers to stepwise or incomplete crystallization. This can also be the result of the heterogeneous nucleation or processing of the material on different days and different crystallization times for the material.

**Figure 5:**
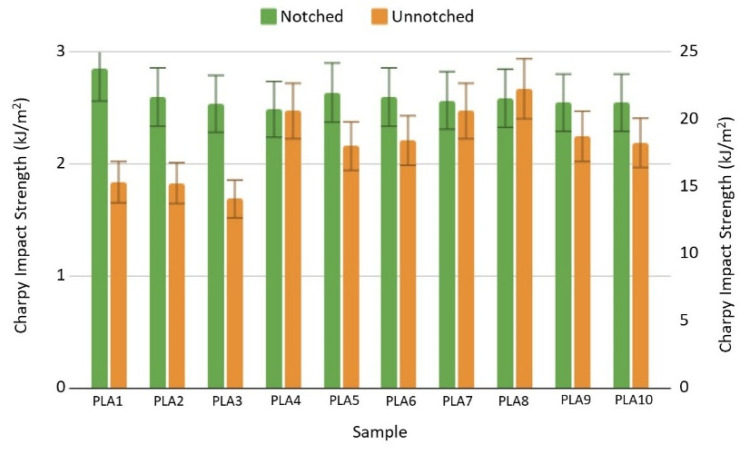
Impact test results for notched (green) and unnotched (orange) bars.

The reaction of the material to a high stress load can be measured by an impact test, resulting in the impact strength and evaluation of the brittleness/ductility of the material. Brittle materials, like PLA, have very low impact strength in both notched and unnotched examined samples. Comparing the impact strength from the first to the tenth cycle within notched samples, the value slightly decreases from 2.85 to 2.55 kJ/m^2^ (Figure 5). Within unnotched samples, sample PLA8 has the highest impact strength of 22.3 kJ/m^2^, while the PLA3 sample has the lowest impact strength of 14 kJ/m^2^. All samples, notched and unnotched, have low energy absorption potential and fragile break behavior with almost no plastic deformation.

Flexural and tensile properties of the material are of the highest importance when it comes to the end-use application. Recycled materials usually do not meet requirements for high-performance use, so they are used for more technical applications. In the case of using multiple recycling cycles for PLA, it can be seen (Table 3) that (considering standard deviation) there are no significant differences between the flexural strength and the strain at strength of the examined samples. This indicates that samples retain rigidity, even after ten cycles of thermal processing.

Tensile test results are summarized in Table 4. Measured values indicate that the material is brittle and rigid (which is also proven with previous DMA and impact test results) and has a very small elongation before break. All the examined samples had a characteristic brittle fracture shape in the stress–strain curve. Differences in tensile strength and strain at break are low and not significant, and it can be concluded that multiple recycling does not affect the tensile properties of PLA. Comparing the values of the flexural and tensile tests, it can be concluded that the material can withstand higher stress loads in the transversal direction than in the longitudinal direction, since a higher force is necessary for the material to break.

**Figure 6:**
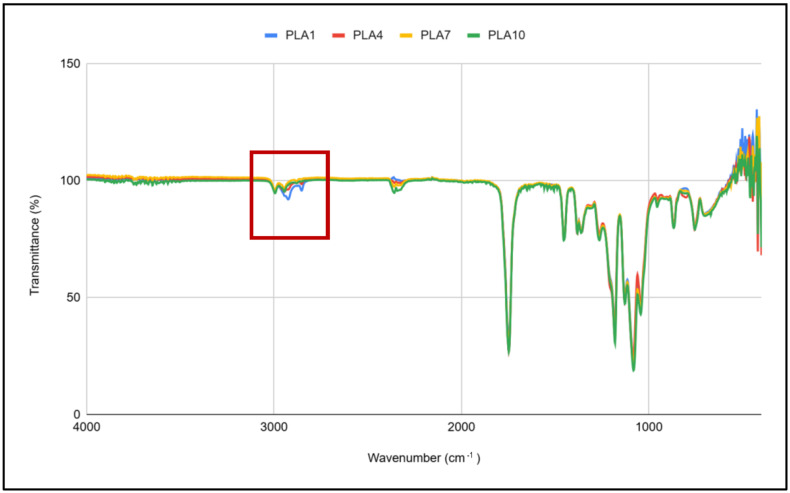
FTIR spectra of selected samples.

**Figure 7:**
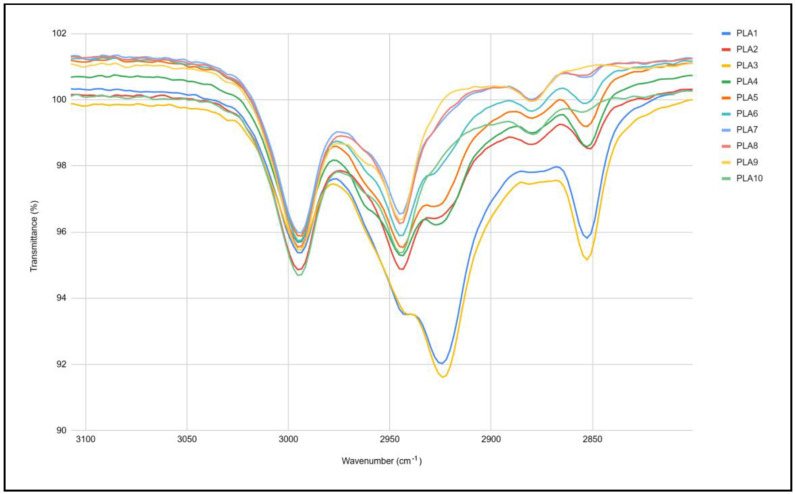
Selection of FTIR peaks.

Changes in the chemical structure of PLA during multiple processing are determined by the FTIR method, and spectra are illustrated in Figure 6 and Figure 7. A difference in peaks around 3000 cm^−1^ was detected, while the difference around 2360–2320 cm^−1^ is related to the CO_2_. The selection of peaks from 3100 to 2800 cm^−1^ is shown in Figure 4. Spectra of PLA1 and PLA3 (bottom blue and yellow lines) have a different shape compared to the others. Spectra of samples PLA9 and PLA10 do not have the peak at 2850 cm^−1^. Peaks from 2995 to 2945 cm^−1^ are related to the CH stretching, and the peak at 2879 cm^−1^ is related to asymmetric -CH_3_ stretching, while the peak at 2852 cm^−1^ corresponds to the symmetric -CH_3_ or -CH_2_ stretching. The difference in those peaks can indicate the polymer chain scission, which is in correlation with MFI results.

**Figure 8:**
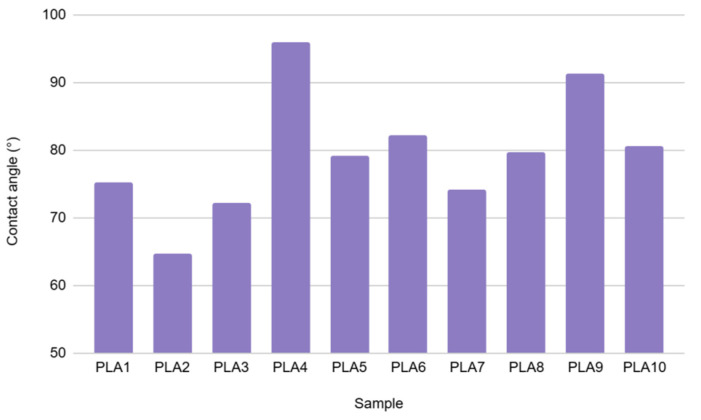
Contact angle of PLA samples.

**Figure 9:**
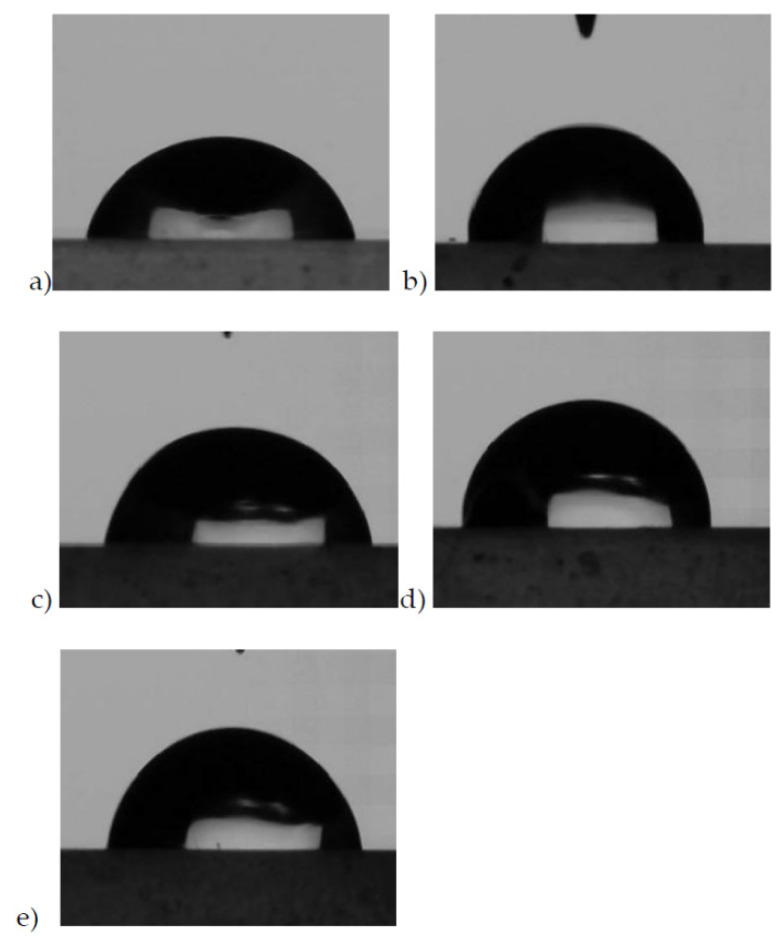
Images of water drops on the surfaces of different samples: (**a**) PLA1, (**b**) PLA4, (**c**) PLA6, (**d**) PLA9, and (**e**) PLA10.

According to the literature [33], PLA has a contact angle between 70° and 80°, which makes its surface vary from moderately hydrophilic to slightly hydrophobic. Values obtained during this experiment fall into this range (Figure 8), except for samples PLA2 (lower value—more hydrophilic), PLA4 and PLA9 (higher value—more hydrophobic). Images of water drops on the sample surface are illustrated in Figure 9. This behavior can also be connected to the different relaxation times for different series of samples, which can cause the differences in surface properties and the orientation of the groups in the polymer chain.

**Figure 10:**
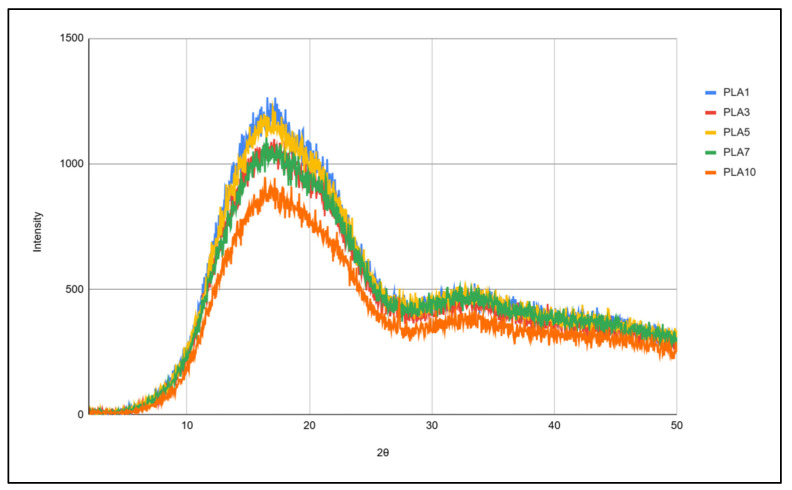
XRD spectra of PLA samples.

The crystallinity of the selected samples is shown in Figure 10. It can be seen that there is no change in the crystallinity of samples within different cycles. There is a wide peak from 10° to 25°, with a maximum at 16.7°, which, according to the literature data [34], is a result of the scattering of PLA. One peak with very low intensity occurs around 34°, and it is not related to PLA, but can be caused by the crystallization of some additive present in the starting material, since, in this work, technical-grade PLA was used (there is no data on the exact composition or the PLA grade used).

The measured values, along with their respective standard deviations, are presented in Table 5. As the number of recycling cycles increased from PLA1 to PLA10, a clear trend in the optical properties of the samples was observed. The L* parameter consistently decreased from 89.598 ± 0.253 (PLA1) to 84.782 ± 0.232 (PLA10). This reduction in L* indicates that the recycled PLA samples became progressively darker with each successive reprocessing step. Changes were also noted along the chromatic axes. The a* values decreased from −5.878 ± 0.101 (PLA1) to −7.366 ± 0.071 (PLA10), signifying a shift towards a more pronounced green color. Conversely, the b* values showed a consistent increase from 7.98 ± 0.112 (PLA1) to 9.848 ± 0.107 (PLA10), indicating an intensified yellow tone. The combined effect of these changes in a* and b* parameters suggests a distinct yellowish-green discoloration of the PLA material upon repeated recycling.

Further analysis of the color saturation, represented by the c* parameter, revealed an increase from 9.912 ± 0.151 (PLA1) to 12.298 ± 0.123 (PLA10). This indicates that the observed color changes are not merely shifts in tone, but also an increase in the vibrancy or intensity of the yellowish-green color. Overall, the colorimetric measurements strongly suggest that the repetitive thermal and mechanical stresses associated with multiple recycling cycles lead to a noticeable degradation of the PLA material, visually manifested as a darkening and a more intense yellowish-green discoloration. These optical changes are likely a direct consequence of polymer degradation mechanisms, such as chain scission and oxidation, which can alter the polymer’s light absorption and scattering properties.

**Figure 11:**
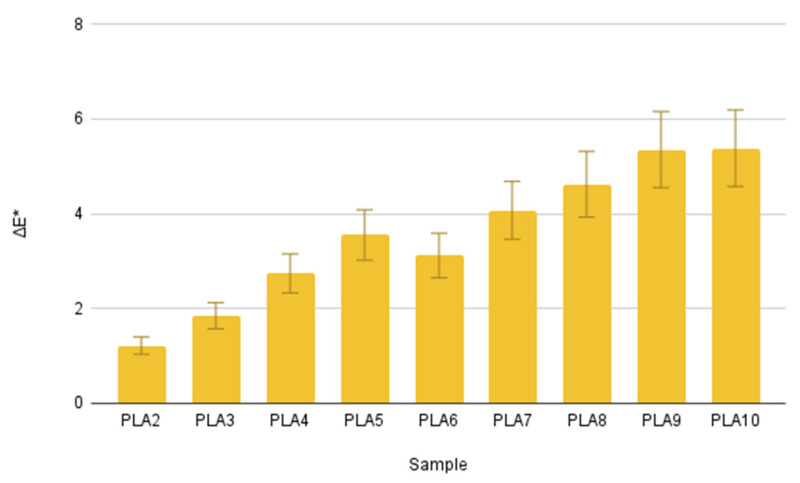
Color changes of the samples.

The color change development from the first to the tenth injection molding cycle is almost linear. Figure 11 illustrates the increase in ΔE* values from the second to the tenth sample, which interprets the sample’s color difference compared to the reference (in this case, PLA1).

## 4. Conclusions

Within this work, we study the effect of additive-free multiple processing of PLA on mechanical, thermal, optical, and chemical properties. It is shown that multiple mechanical recycling of pre-consumer PLA waste, consisting of 10 cycles of injection molding and milling, does not significantly change the properties of the resulting PLA compared to the starting material without the use of additives or virgin material.

An increase in MFI from 8.7 to 13.9 was observed, which indicates a decrease in molecular weight, and since no additives were added, it is a direct consequence of chain scission and a decrease in the molecular weight due to the successive processing.

The key mechanical properties, the tensile strength, flexural strength, and modulus, were not affected by reprocessing, which is also the consequence of the shorter chains present, since the packing and the mobility of the chains was better.

Thermal properties remained stable accross cycles, which indicates that thermal stability is not affected by the multiple processing of the material; however, due to the shorter chain present, crystallization of PLA was facilitated.

Changes in the contact angle of the PLA surface are consequences of the orientation and packaging of the molecules within item, but for most of the samples, this remained within a theoretical range of values.

Crystallinity of PLA was not affected with multiple processing; the characteristic peak of crystallinity remained at the same 2θ value.

The main drawback of recycling is the color change that develops from the first to the tenth cycle, making the material more yellow and dull. This color change, driven by polymer degradation mechanisms such as chain scission and oxidation, may limit the reuse of a material in esthetically sensitive applications unless color stabilization strategies are introduced.

Overall, these findings reinforce the feasibility of the closed-loop mechanical recycling of PLA industrial waste for sustainable material management, providing valuable insights for advancing circular economy practices in the processing of bioplastics.

Further investigation of the recycling of PLA items after service life together with biodegradability and compostability studies could fulfill the obtained results.

## Figures and Tables

**Table 1:** Thermal data from second heating cycle.

Sample	Tg (°C)	Tcc (°C)	ΔHcc (J/g)	Tm (°C)	ΔHm (J/g)	Xc (%)	Td_1_ (°C)
Milled PLA	59.5	113.1	17.6	148.9	26.0	9.0	370.1
Extruded PLA	59.3	114.4	22.2	148.8	23.2	1.1	369.0
PLA1	59.2	114.4	23.2	148.7	24.1	1.0	368.7
PLA2	59.9	115.2	16.8	149.7	28.8	12.9	368.5
PLA3	59.7	115.2	16.4	149.8	28.7	13.2	368.1
PLA4	59.5	114.7	16.9	148.9	28.3	12.1	367.5
PLA5	59.5	115.0	24.5	149.6	25.5	1.1	366.9
PLA6	59.7	114.2	18.7	149.2	28.4	10.3	367.1
PLA7	59.4	114.5	17.2	148.4	27.9	11.4	366.6
PLA8	59.4	114.0	17.7	148.5	27.6	10.5	364.8
PLA9	59.7	114.4	17.3	149.2	27.9	11.3	365.3
PLA10	59.5	114.9	23.2	149.4	25.2	2.2	363.7

**Table 2:** Results of DMA analysis of PLA samples.

Sample	E’ @ 30 °C (GPa)	tanδ	Tg	tanδ cc (-)	tanδ cc (°C)	E’ cc (GPa)
PLA1	2.86	1.94	66.96	95.10	0.17	0.11
PLA2	2.95	1.90	67.28	95.30	0.16	0.10
PLA3	2.94	1.97	67.56	95.70	0.17	0.09
PLA4	3.00	1.99	67.48	94.50	0.16	0.10
PLA5	2.78	1.97	67.44	95.60/107.70	0.17/0.13	0.07/0.08
PLA6	2.86	1.93	66.64	95.10/109.20	0.16/0.09	0.10/0.09
PLA7	2.92	1.97	66.72	95.60/105.20/115.30	0.16/0.12/0.11	0.08/0.07
PLA8	3.01	1.99	66.76	95.40/106.60	0.16/0.14	0.07/0.08
PLA9	2.80	1.96	66.40	93.30	0.15	0.10
PLA10	2.68	2.05	67.19	94.30	0.17	0.10

**Table 3:** Flexural test results.

Sample	Flexural Modulus (GPa)	Flexural Strength (MPa)	Strain at Strength (%)
PLA1	3.25 ± 0.01	94.41 ± 0.05	4.25 ± 0.05
PLA2	3.23 ± 0.05	94.19 ± 0.25	4.24 ± 0.02
PLA3	3.30 ± 0.07	94.50 ± 0.63	4.29 ± 0.09
PLA4	3.28 ± 0.08	98.59 ± 0.32	4.35 ± 0.02
PLA5	3.32 ± 0.03	93.87 ± 0.84	4.10 ± 0.17
PLA6	3.31 ± 0.01	93.78 ± 0.44	4.21 ± 0.04
PLA7	3.33 ± 0.03	94.59 ± 0.43	4.14 ± 0.13
PLA8	3.34 ± 0.05	95.27 ± 0.47	4.25 ± 0.05
PLA9	3.44 ± 0.03	97.31 ± 1.58	4.08 ± 0.40
PLA10	3.39 ± 0.05	98.05 ± 0.26	4.30 ± 0.06

**Table 4:** Tensile test results.

Sample	Tensile Modulus (GPa)	Tensile Strength (MPa)	Strain at Strength (%)	Strain at Break (%)
PLA1	2.62 ± 0.23	68.20 ± 2.72	4.58 ± 0.29	5.18 ± 0.78
PLA2	2.55 ± 0.26	67.40 ± 0.51	4.48 ± 0.36	5.72 ± 0.55
PLA3	2.66 ± 0.33	67.90 ± 1.16	4.36 ± 0.35	5.34 ± 0.61
PLA4	2.00 ± 0.25	69.00 ± 0.73	4.22 ± 0.16	5.92 ± 0.90
PLA5	3.18 ± 0.15	67.50 ± 0.85	4.15 ± 0.24	4.94 ± 0.49
PLA6	2.69 ± 0.38	66.90 ± 0.92	4.08 ± 0.15	5.79 ± 0.64
PLA7	3.21 ± 0.32	66.60 ± 0.71	4.13 ± 0.15	4.99 ± 0.16
PLA8	2.73 ± 0.15	65.50 ± 0.74	4.13 ± 0.08	5.04 ± 0.37
PLA9	2.60 ± 0.33	66.30 ± 0.34	4.19 ± 0.15	5.67 ± 0.62
PLA10	2.86 ± 0.23	67.10 ± 0.8	4.09 ± 0.09	5.30 ± 0.41

**Table 5:** Colorimetric values of examined samples.

Sample	L*	a*	b*	c*	h*
PLA1	89.60 ± 0.25	−5.88 ± 0.10	7.98 ± 0.112	9.91 ± 0.15	126.38 ± 0.11
PLA2	88.51 ± 0.31	−6.22 ± 0.13	8.43 ± 0.13	10.48 ± 0.18	126.40 ± 0.19
PLA3	87.96 ± 0.50	−6.42 ± 0.19	8.64 ± 0.23	10.76 ± 0.30	126.61 ± 0.17
PLA4	87.17± 0.32	−6.67 ± 0.11	8.97 ± 0.16	11.17 ± 0.19	126.66 ± 0.14
PLA5	86.43 ± 0.20	−6.91 ± 0.09	9.22 ± 0.16	11.52 ± 0.17	126.84 ± 0.31
PLA6	86.80 ± 0.13	−6.77 ± 0.07	9.06 ± 0.08	11.31 ± 0.09	126.76 ± 0.27
PLA7	85.98 ± 0.28	−7.02 ± 0.10	9.45 ± 0.11	11.77 ± 0.14	126.57 ± 0.23
PLA8	85.47 ± 0.66	−7.17 ± 0.22	9.60 ± 0.24	11.98 ± 0.32	126.75 ± 0.20
PLA9	84.81 ± 0.69	−7.38 ± 0.24	9.84 ± 0.30	12.3 ± 0.38	126.87 ± 0.07
PLA10	84.78 ± 0.23	−7.37 ± 0.07	9.85 ± 0.11	12.30 ± 0.12	126.80 ± 0.17

## Data Availability

The dataset is available on request from the authors (alexm@uns.ac.rs, rebeka.lorber@ftpo.eu).

## Abbreviations

The following abbreviations are used in this manuscript:

PLA	Poly(lactide)
EoL	End-of-Life
MFI	Melt flow index
DSC	Differential scanning calorimetry
Tc	Crystallization temperature
ΔHc	Crystallization enthalpy
Tm	Melting temperature
ΔHm	Melting enthalpy
Xc	Relative crystallinity
ΔHf	Metling enthalpy of 100% crystalline PLA
TGA	Thermo-gravimetric analysis
DMA	Dynamic-mechanical analysis
FTIR	Infrared spectroscopy with Fourrier transformation
XRD	X-ray diffraction
Td_1_	Degradation temperature
Tg	Glass transition temperature
